# Deep learning segmentation architectures for automatic detection of pancreatic ductal adenocarcinoma in EUS-guided fine-needle biopsy samples based on whole-slide imaging

**DOI:** 10.1097/eus.0000000000000094

**Published:** 2024-12-12

**Authors:** Anca Loredana Udriștoiu, Nicoleta Podină, Bogdan Silviu Ungureanu, Alina Constantin, Claudia Valentina Georgescu, Nona Bejinariu, Daniel Pirici, Daniela Elena Burtea, Lucian Gruionu, Stefan Udriștoiu, Adrian Săftoiu

**Affiliations:** 1 Faculty of Automation, Computers and Electronics, University of Craiova, Craiova, Romania; 2 Department of Gastroenterology, Ponderas Academic Hospital, Bucharest, Romania; 3 Faculty of Medicine, Carol Davila University of Medicine and Pharmacy, Bucharest, Romania; 4 Department of Gastroenterology, University of Medicine and Pharmacy of Craiova, Craiova, Romania; 5 Research Center of Gastroenterology and Hepatology, University of Medicine and Pharmacy Craiova, Craiova, Romania; 6 Department of Pathology, Clinical Emergency County Hospital of Craiova, Craiova, Romania; 7 REGINA MARIA Regional Laboratory, Pathological Anatomy Division, Cluj-Napoca, Romania; 8 Department of Histology, University of Medicine and Pharmacy of Craiova, Craiova, Romania; 9 Faculty of Mechanics, University of Craiova, Craiova, Romania; 10 Department of Gastroenterology and Hepatology, Elias University Emergency Hospital, Carol Davila University of Medicine and Pharmacy, Bucharest, Romania.

**Keywords:** EUS-guided fine-needle biopsy, Pancreatic ductal adenocarcinoma, Artificial intelligence, Deep learning, Whole-slide imaging

## Abstract

**Background:**

EUS-guided fine-needle biopsy is the procedure of choice for the diagnosis of pancreatic ductal adenocarcinoma (PDAC). Nevertheless, the samples obtained are small and require expertise in pathology, whereas the diagnosis is difficult in view of the scarcity of malignant cells and the important desmoplastic reaction of these tumors. With the help of artificial intelligence, the deep learning architectures produce a fast, accurate, and automated approach for PDAC image segmentation based on whole-slide imaging. Given the effectiveness of U-Net in semantic segmentation, numerous variants and improvements have emerged, specifically for whole-slide imaging segmentation.

**Methods:**

In this study, a comparison of 7 U-Net architecture variants was performed on 2 different datasets of EUS-guided fine-needle biopsy samples from 2 medical centers (31 and 33 whole-slide images, respectively) with different parameters and acquisition tools. The U-Net architecture variants evaluated included some that had not been previously explored for PDAC whole-slide image segmentation. The evaluation of their performance involved calculating accuracy through the mean Dice coefficient and mean intersection over union (IoU).

**Results:**

The highest segmentation accuracies were obtained using Inception U-Net architecture for both datasets. PDAC tissue was segmented with the overall average Dice coefficient of 97.82% and IoU of 0.87 for Dataset 1, respectively, overall average Dice coefficient of 95.70%, and IoU of 0.79 for Dataset 2. Also, we considered the external testing of the trained segmentation models by performing the cross evaluations between the 2 datasets. The Inception U-Net model trained on Train Dataset 1 performed with the overall average Dice coefficient of 93.12% and IoU of 0.74 on Test Dataset 2. The Inception U-Net model trained on Train Dataset 2 performed with the overall average Dice coefficient of 92.09% and IoU of 0.81 on Test Dataset 1.

**Conclusions:**

The findings of this study demonstrated the feasibility of utilizing artificial intelligence for assessing PDAC segmentation in whole-slide imaging, supported by promising scores.

## INTRODUCTION

Pancreatic ductal adenocarcinoma (PDAC) ranks as the fourth leading cause of cancer-related deaths in the United States, with a grim 5-year relative survival rate of only 12%.^[1]^ Key risk factors include family history, smoking, male gender, obesity, and type 2 diabetes, with rising incidence rates reflective of increasing obesity and diabetes prevalence. Often presenting with nonspecific symptoms such as fatigue and jaundice, most patients are diagnosed at advanced stages, with only 15%–20% eligible for potentially curative surgery due to late detection.^[2,3]^

Early detection is crucial for improving prognosis, as small, localized tumors offer a better chance for survival. EUS has emerged as the preferred method for early diagnosis and staging, offering high-resolution images superior to computed tomography (CT) or magnetic resonance imaging (MRI) scans. EUS-related techniques, such as contrast-enhanced EUS and elastography, alongside EUS-guided tissue acquisition methods such as fine-needle aspiration (FNA) biopsy, have improved diagnostic accuracy, especially when backed by artificial intelligence (AI) models.^[4,5]^ Despite these advances, the histological diagnosis remains challenging due to PDAC’s complex growth patterns and similarities to chronic pancreatitis.^[6]^

The introduction of AI techniques, particularly deep learning models, has revolutionized the pathological diagnosis process.^[7]^ Deep learning enhances the prediction and segmentation of cancerous tissue in whole-slide images (WSIs), utilizing encoder-decoder network structures such as the U-Net architecture. U-Net and its variants, such as Attention U-Net, Residual U-Net, Inception U-Net, Dense U-Net, Squeeze and Excite U-Net, and U-Net++, have shown promise in segmenting PDAC tissue with high precision.^[8–19]^

Our study compares 7 U-Net variants across 2 PDAC WSI datasets from different populations, aiming to evaluate their segmentation performance, computational efficiency, and complexity. This comprehensive comparison seeks to identify the most effective architectural designs for PDAC WSI segmentation, contributing to the field’s understanding and aiding in the early detection and diagnosis of this deadly cancer.

## MATERIAL AND METHODS

### Datasets and annotation

In this study, we used 2 different datasets of EUS-guided fine-needle biopsy (FNB) samples from 2 medical laboratories (Craiova and Bucharest), further labeled as Dataset 1 and Dataset 2.

The tissue samples from the 2 laboratories were fixed in 10% neutral buffered formalin, routinely processed for paraffin embedding, then cut as 4-μm-thick sections, stained with hematoxylin and eosin, and cover slipped with a xylene-based mounting medium, DPX (Sigma-Aldrich, St Louis, MO).

The whole slides from Dataset 1 were scanned on a Motic EasyScan slide scanner (Motic Group Co, Ltd, Xiamen, China), at 20× magnification (0.52 μm/pixel), and images were saved in the uncompressed proprietary format.

The whole slides from Dataset 2 were scanned on APERIO LV1 IVD slide scanner (Leica) under the same acquisition conditions with a magnification of 20× (0.275 μm/pixel), and images were saved in the uncompressed proprietary format.

**Figure 1:**
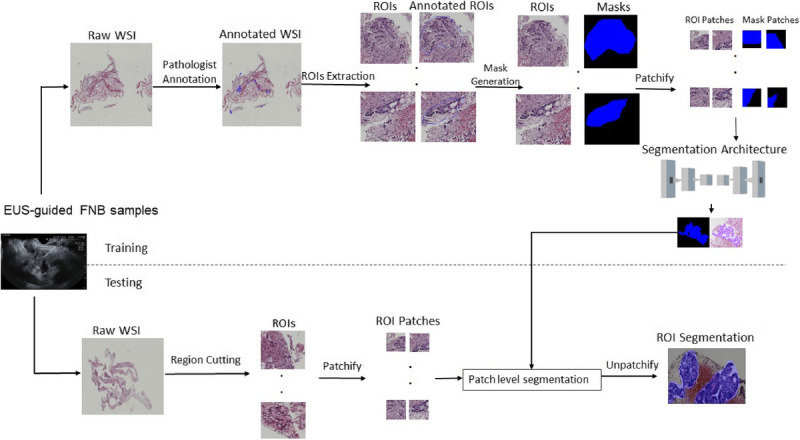
The steps for semantic segmentation of PDAC WSIs. PDAC, pancreatic ductal adenocarcinoma; WSI: Whole-slide imaging.

The obtained WSIs were multigigabyte images with typical resolutions of 40,000 × 40,000 pixels, although each WSI had a different size. The large input image dimensions would have raised the count of parameters to be estimated, along with the necessary computational power and memory. So, in our study, we extracted regions of interest (ROIs) from WSIs at high magnification. The workflow for the deep learning segmentation of PDAC WSIs was described in Figure 1.

The creation of the 2 datasets involved the following procedures:

(1) Data Annotation

**Figure 2:**
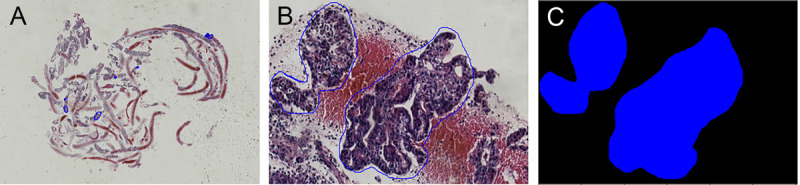
The process of WSI annotation and extraction of ROIs. A, The WSI annotated by pathologist. B, An example of an ROI manually extracted from the annotated WSI. C, The mask generation for the extracted ROI. ROI: Region of interest; WSI: Whole-slide imaging.

In this step, the pathologists examined WSIs at high resolution and identified the regions containing PDAC histological information [Figure 2A]. These identified regions, referred to as ROIs, served as input for subsequent stages. The pathologist’s task was to annotate these ROIs, as they constituted the data used for training and validating models.

(2) ROI extraction and mask generation

Each PDAC region of interest was annotated by a pathologist using a blue line for contouring as in Figure 2A. Then, we manually extracted the bounding box of each annotated region as in Figure 2B. The mask was generated for each ROI, and the positive PDAC region was colored deep blue, whereas the negative region was colored black as in Figure 2C.

(3) Patch extraction and datasets construction

Patches were subsampled into 256 × 256 pixels with a half overlap ratio from extracted PDAC ROIs. Utilizing smaller patches enabled our model to effectively capture the PDAC subtle features. Patches were included in both datasets (Dataset 1 and Dataset 2), if they included a PDAC surface. These patches were used to train and evaluate the segmentation U-Net models.

Dataset 1 contained 31 PDAC WSIs from which we extracted 4040 small patches of 256 × 256 pixels size as in Table 1. A number of 2940 patches extracted from 26 WSIs were used for training (2228 patches) and validation (712 patches). A number of 1100 patches extracted from 5 WSIs were used for testing.

**Table 1:** Dataset 1 split into training, validation, and test datasets.

Dataset	Training	Validation	Test	Total
No WSIs	26		5	31
No patches	2228	712	1100	4040

WSI: Whole-slide imaging.

Dataset 2 contained 33 PDAC WSIs from which we extracted 4294 small patches of 256 × 256 pixels size as in Table 2. A number of 3094 patches extracted from 26 WSIs were used for training (2,294 patches) and validation (800 patches). One thousand two hundred patches extracted from 7 WSIs were used for testing.

**Table 2:** Dataset 2 split into training, validation, and test datasets.

Dataset	Training	Validation	Test	Total
No WSIs	26		7	33
No patches	2294	800	1200	4294

WSI: Whole-slide imaging.

### Segmentation models architecture and parameters

All networks were trained to perform semantic segmentation of PDAC WSIs as in Figure 1. The output of each network consisted of class probabilities corresponding to each pixel in the original input PDAC image. The probabilities represented the classification of each pixel into 1 of the 2 regions (positive and negative) of the PDAC images.

For comparison, all 7 architectures^[20–27]^ included:

- Three convolutions per layer
- Max pooling layers (2 × 2) for downsampling and transposed convolutions for upsampling (2 × 2 kernel and strides)
- Four pooling/upsampling layers with 16 filters for all convolutions in the first layer, which was doubled after each pooling and subsequently halved at each upsampling layer
- Each layer, except for the output layer, was followed by batch normalization^[28]^ and a rectified linear unit activation.
- During the training phase, the Adam optimizer^[29]^ was utilized with default parameters.
- All networks were trained for 100 epochs with a batch size of 16 images.
- We calculated the gradient of Dice loss in backpropagation, as in Equations (1), (2).

For models’ selection, we used the epoch with the best Dice coefficient on the validation sets. The Dice coefficient was a measure of similarity between 2 samples (in this case, the predicted segmentation mask and ground truth mask) and was defined as in Equation (1):


$$\text{Dice}=\frac{2*\mathrm{TP}}{2*\mathrm{TP}+\mathrm{FP}+\mathrm{FN}}$$ (1)

Dice loss=1−Dice (2)

where TP was the number of true positives, FP was the number of false positives, and FN was the number of false negatives.

We performed on-the-fly augmentation during training, meaning that augmented patches were generated in real time. This ensured variability throughout training by making the model see new data every epoch. To not distort the unique information of WSI patches, we used only random flips along horizontal or vertical axes to increase the diversity of spatial patterns of patches.

The evaluation of different architectures involved comparing the Dice coefficient and mean intersection over union (IoU) across each testing set, using the model chosen for its performance from the best epoch.

The IoU was computed as in Equation (3):


$$\mathrm{IoU}=\frac{\text{Area of intersection}}{\text{Area of union}}$$ (3)

where the “area of intersection” was the area where our predicted mask overlapped the ground truth mask, and the “area of union” was the area combining our predicted mask and the ground truth mask.

Following training, predictions on the test sets were generated by first extracting regions of 1024 × 1024 pixels from WSIs. Subsequently, these regions were divided (patchified) into images of 256 × 256 pixels, and the trained models were then applied for patch-level segmentation [Figure 1]. All adjacent segmented patches were recombined (unpatchified) to form a complete ROI prediction. Subsequently, the segmented ROIs were overlaid onto the entire slide image.

Our experiments were undertaken using Tensorflow 2.10 in Python 3.9 using an NVIDIA RTX graphics processing unit.

## EXPERIMENTAL RESULTS

The 7 U-Net architecture variants evaluated in this article are Inception U-Net, Vanilla U-Net, Dense U-Net, Attention U-Net, U-Net++, Residual U-Net, and SE U-Net. The performance was evaluated by considering the accuracy as the mean Dice coefficient and mean IoU, whereas mean epoch training time, mean evaluation time, and the number of parameters showed by comparison the computational complexity of each segmentation model. The following results were obtained for the 2 datasets [Table 3 and Table 4].

**Table 3:** Comparison of results for Test Dataset 1 using the models trained on Train Dataset 1.

Network architecture	Accuracy, %	Mean IoU	Mean epoch train time, s	Mean image evaluation time, ms	Model parameters, ×10**^6^**
Vanilla U-Net	93.68	0.69	51	131	2.93
Attention U-Net	92.72	0.68	49	160	2.97
Dense U-Net	96.43	0.68	71	162	5.4
Inception U-Net	97.82	0.87	91	182	4.6
U-Net++	97.05	0.82	97	175	2.4
Residual U-Net	94.59	0.72	45	135	2.9
SE-U-Net	89.09	0.44	59	152	3.04

IoU: Intersection over union.

**Table 4:** Comparison of results for Test Dataset 2 using the models trained on Train Dataset 1.

Network architecture	Accuracy, %	Mean IoU	Mean epoch train time, s	Mean image evaluation time, ms	Model parameters, ×10**^6^**
Vanilla U-Net	94.72	0.74	53	143	2.93
Attention U-Net	87.75	0.55	59	165	2.97
Dense U-Net	94.66	0.74	76	168	5.4
Inception U-Net	95.70	0.79	96	186	4.6
U-Net++	85.78	0.51	103	186	2.4
Residual U-Net	91.52	0.64	48	146	2.9
SE-U-Net	83.44	0.47	64	169	3.04

IoU: Intersection over union.

The results suggested that the Inception U-Net model, which had increased complexity and greatest evaluation time, performed best for both datasets, recording an accuracy of 97.82% and an average IoU of 0.87 for Dataset 1, respectively, an accuracy of 95.70%, and an average IoU of 0.79 for Dataset 2. The fastest to train and evaluate, Vanilla U-Net performed well in terms of accuracy and IoU for both datasets (93.68% accuracy and 0.69 IoU for Dataset 1, respectively, 94.72% and 0.74 IoU for Dataset 2). The performance between the 2 datasets varies for the 7 segmentation models, due to the high complexity, subjectivity, and quality of histological images. Dataset 1 was a relatively small dataset, and all cases were mostly well-differentiated, allowing manual delineation. Dataset 2 contained more complex cases, where tumors with complex and reticular growth patterns were difficult to be accurately annotated.

In the external testing, the Test Dataset 1 was used to evaluate the best 3 segmentation models (Inception U-Net, Vanilla U-Net, Dense U-Net) trained on Train Dataset 2 [Table 5], and, respectively, the Test Dataset 2 was used to evaluate the best 3 segmentation models (Inception U-Net, U-Net++, Dense U-Net) trained on Train Dataset 1 [Table 6]. The results of the external testing are shown in Table 5 and Table 6. A decline in accuracy metric was observed, primarily due to the complexity and differences between the 2 datasets (Test Dataset 1 and Test Dataset 2).

**Table 5:** Results of external testing on Test Dataset 2 using the models trained on Train Dataset 1.

Network architecture	Accuracy, %	Mean IoU
Dense U-Net	90.52	0.65
U-Net++	91.99	0.70
Inception U-Net	93.12	0.74

IoU: Intersection over union.

**Table 6:** Results of external testing on Test Dataset 1 using the models trained on Train Dataset 1.

Network architecture	Accuracy, %	Mean IoU
Vanilla U-Net	90.10	0.77
Dense U-Net	91.89	0.80
Inception U-Net	92.09	0.81

IoU: Intersection over union.

**Figure 3:**
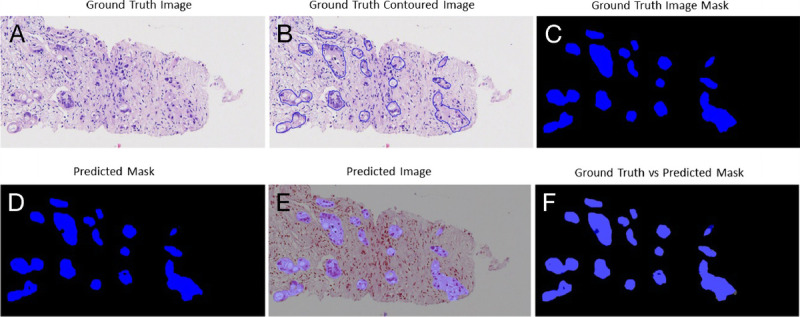
Segmentation results for an image from Test Dataset 1 using the Inception U-Net model trained on Dataset 1: (A) ground truth image; (B) the original contoured image; (C) ground truth mask; (D) predicted segmentation mask; (E) the predicted segmentation mask superimposed over the original image; (F) differences between the ground truth mask and the predicted mask.

**Figure 4:**
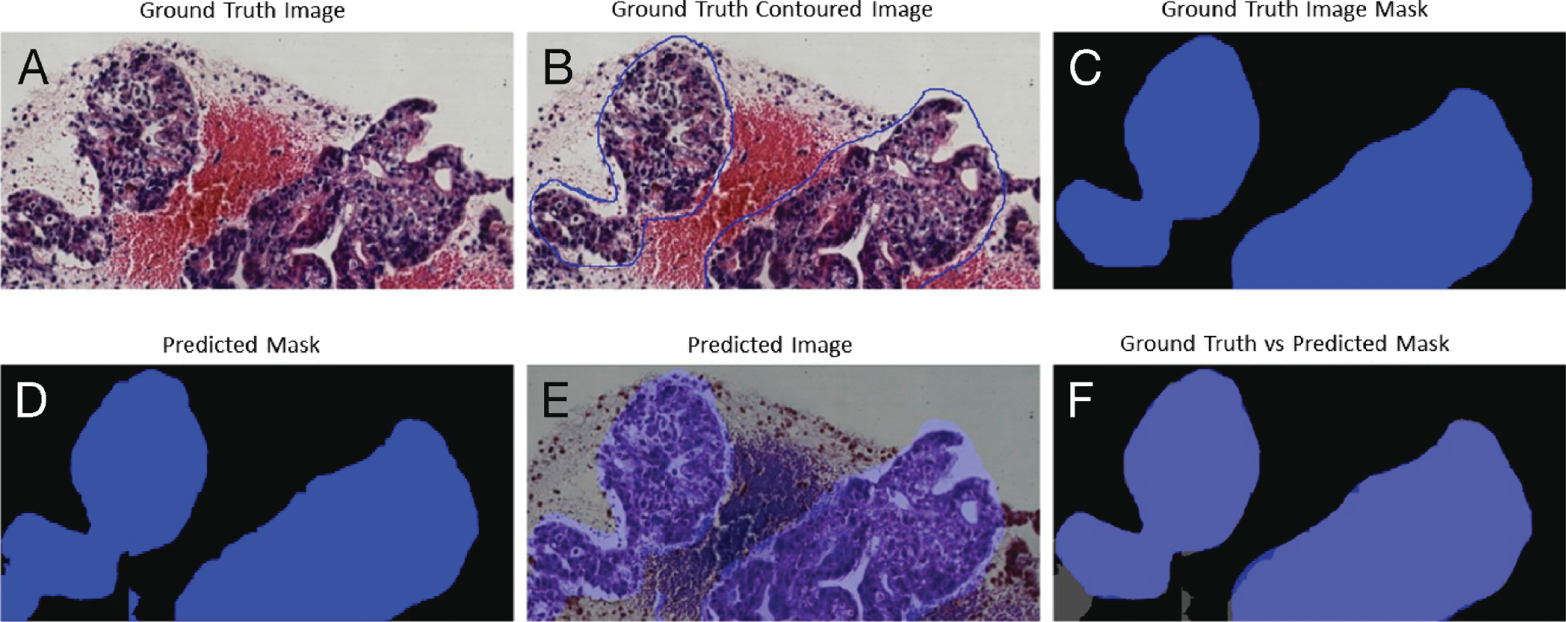
Segmentation results for an image from Test Dataset 2 using the Inception U-Net model trained on Dataset 2: (A) ground truth image; (B) the original contoured image; (C) ground truth mask; (D) predicted mask; (E) predicted mask superimposed over the original image; (F) differences between ground truth mask and predicted mask.

**Figure 5:**
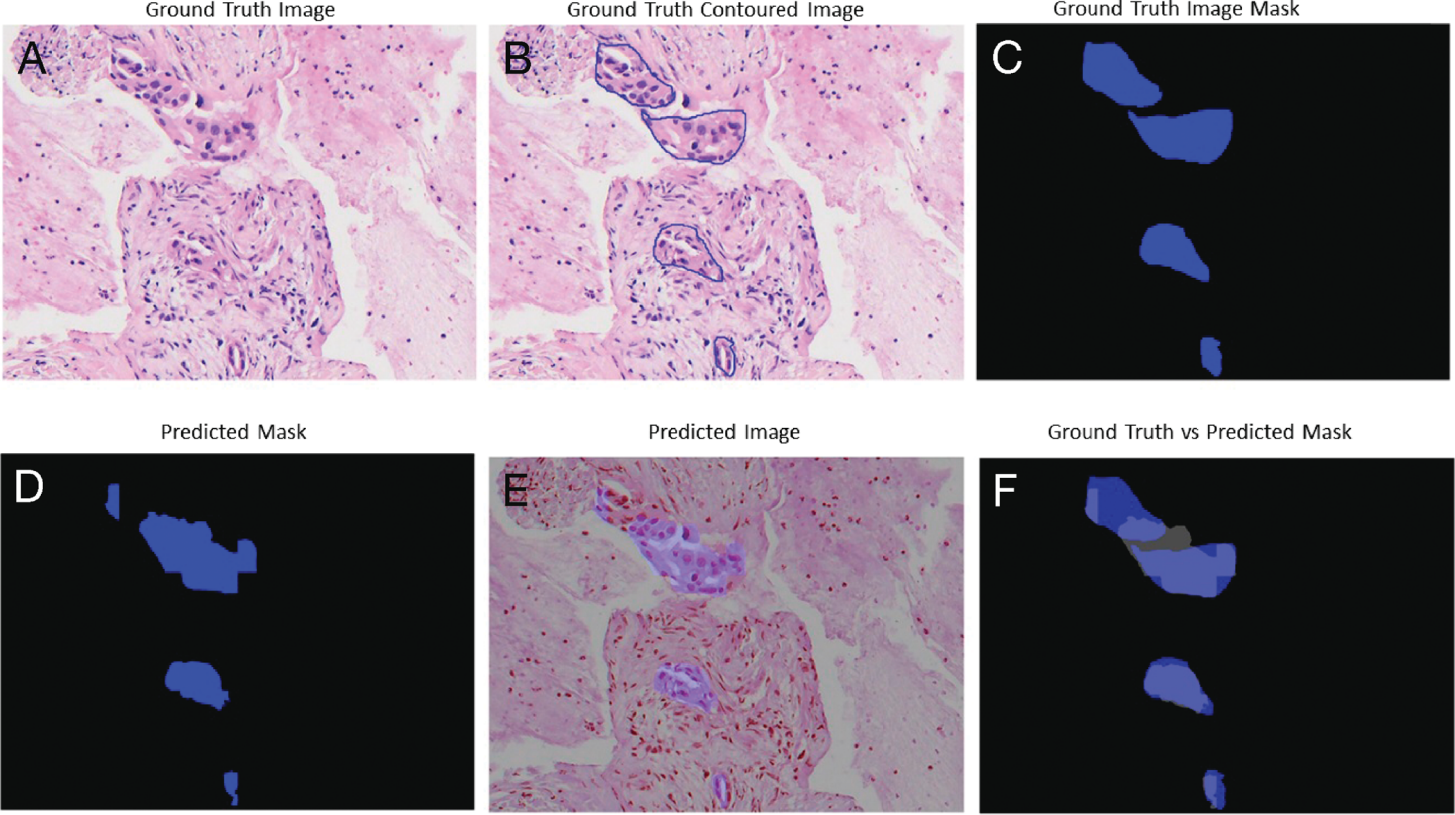
Segmentation results for an image from Test Dataset 1 using the Vanilla U-Net model trained on Dataset 1: (A) ground truth image; (B) the original contoured image; (C) ground truth mask; (D) predicted segmentation mask; (E) the predicted segmentation mask superimposed over the original image; (F) differences between the ground truth mask and the predicted mask.

**Figure 6:**
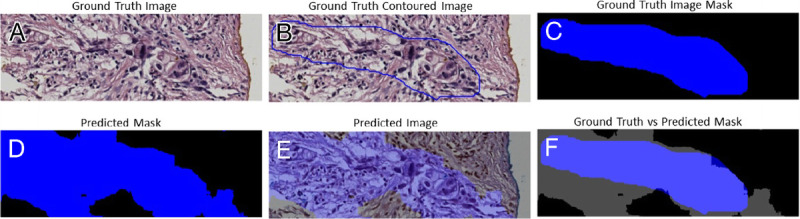
Segmentation results for an image from Test Dataset 2 using the Vanilla U-Net model trained on Dataset 2: (A) ground truth image; (B) the original contoured image; (C) ground truth mask; (D) predicted segmentation mask; (E) the predicted segmentation mask superimposed over the original image; (F) differences between the ground truth mask and the predicted mask.

The trade-off between performances, complexity, and speed was important to be considered for histological samples of very large dimensions, which required time and hardware. Figures 3, 4, 5, and 6 gave some example segmentation outputs by comparison to the ground truth examples for each of the 2 datasets using the trained Inception U-Net models, respectively, the trained Vanilla U-Net models. The results showed the robustness and accuracy of the trained segmentation models.

In Figure 3, a patch-level segmentation on an image example from Test Dataset 1 using the Inception U-Net model trained on Dataset 1 is shown. Figure 3A shows an original sample ROI extracted from a WSI, Figure 3B shows the PDAC original contoured image, and Figure 3C shows the PDAC original mask with the positive PDAC area colored deep blue. Figure 3D shows the predicted segmentation mask colored deep blue. Figure 3E shows the predicted segmentation mask superimposed over the original image. Figure 3F shows the difference between the original ground truth mask and predicted segmentation mask. The intersection between the 2 masks was colored light blue. We can observe in Figure 3F that certain small regions colored deep blue were wrongly predicted as false negative, whereas other small regions colored gray were wrongly predicted as false positive.

In Figure 4, a patch-level segmentation on an image example from Test Dataset 2 using Inception U-Net model trained on Dataset 2 is shown. Figure 4A shows an original sample ROI extracted from a WSI, Figure 4B shows the PDAC original contoured image, and Figure 4C shows PDAC original mask with 2 positive PDAC areas colored deep blue. Figure 4D shows the predicted mask colored deep blue. Figure 4E shows the predicted mask superimposed over to the original image. Figure 4F shows the difference between the original ground truth mask and predicted mask. The area where the 2 masks intersected was colored light blue. We can observe in Figure 4F that certain small regions colored deep blue were wrongly predicted as false negative, and 2 small gray regions were wrongly predicted as false positive.

In Figure 5, a patch-level segmentation on an image example from Test Dataset 1 using the Vanilla U-Net model trained on Dataset 1 is shown.

In Figure 6, a patch-level segmentation on an image example from Test Dataset 2 using Vanilla U-Net model trained on Dataset 2 is shown.

**Figure 7:**
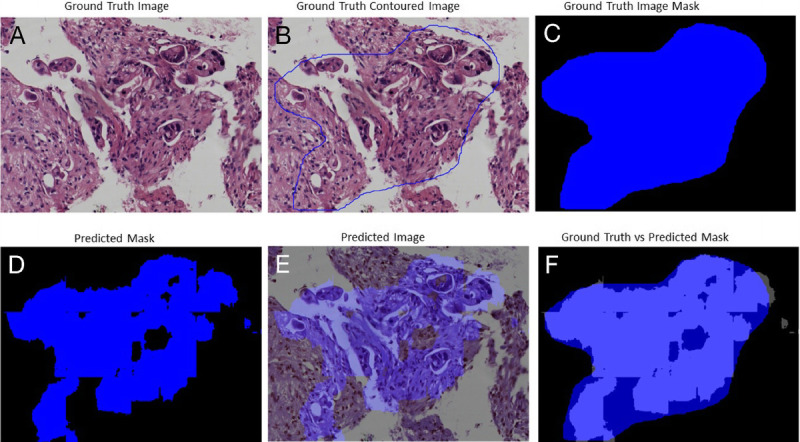
Segmentation results for an image from Test Dataset 2 using the Inception U-Net model trained on Dataset 1: (A) ground truth image; (B) the original contoured image; (C) ground truth mask; (D) predicted segmentation mask; (E) the predicted segmentation mask superimposed over the original image; (F) differences between the ground truth mask and the predicted mask.

**Figure 8:**
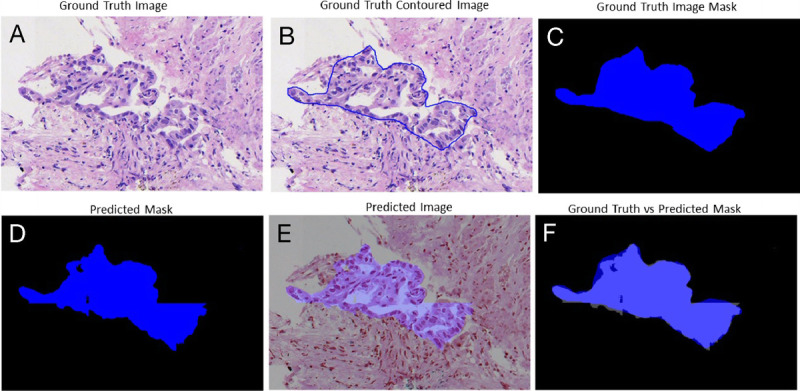
Segmentation results for an image from Test Dataset 1 using the Inception U-Net model trained on Dataset 2: (A) ground truth image; (B) the original contoured image; (C) ground truth mask; (D) predicted segmentation mask; (E) the predicted segmentation mask superimposed over the original image; (F) differences between the ground truth mask and the predicted mask.

In Figure 7 and Figure 8, the external testing examples of patch-level segmentation on an image example from Test Dataset 2 using the Inception U-Net model trained on Dataset 1 and, respectively, on an image example from Test Dataset 1 using the Inception U-Net model trained on Dataset 2 are shown. Both trained models demonstrated accurate patch segmentation, with the model trained on Dataset 2 achieving a particularly high IoU.

## DISCUSSION

Research in AI-assisted detection of pancreatic cancer primarily focuses on imaging modalities such CT and MRI, but also EUS.^[30]^ For CT imaging, Panda et al. proposed a 2-stage 3-dimensional Convolution Neural Networks (CNN) model based on a modified U-Net architecture, achieving an average Dice similarity coefficient of 91% on portal venous phase CT scans.^[31]^ Likewise, Abel et al. focused on detecting pancreatic cystic lesions using a 2-step nnU-Net architecture, obtaining a mean sensitivity of 78.8% with higher sensitivity for larger and distally located lesions.^[32]^ For MRI, Chen et al. utilized a modified faster R-CNN for classifying cystic pancreatic neoplasms, reaching a patient-level accuracy of 92.3%.^[33]^

For EUS, Zhang et al. developed models for station classification and pancreas segmentation using EUS images, achieving accuracies of 94.2% and 82.4% in internal and external validations, respectively, with their segmentation model recording a Dice coefficient of 0.836 internally and 0.715 externally.^[34]^ Moreover, Iwasa and colleagues used the U-Net architecture to detect tumors in contrast-enhanced EUS video images, training their model on data from 100 patients and achieving a median IoU of 0.77.^[35]^

AI’s role extends to analyzing WSIs and supporting the diagnostic process of fine-needle aspiration (FNA) and FNB samples.^[36–39]^ Kriegsmann et al. trained an efficient net architecture on histopathological slides for classifying pancreatic tissue types,^[40]^ whereas Niazi et al. developed a CNN for differentiating between neuroendocrine tumors and nontumor regions in Ki67-stained biopsy images, achieving high sensitivity and specificity.^[41]^

For cytology, Momeni-Boroujeni et al. used a K-means clustering algorithm and a multilayer perceptron neural network to distinguish between benign and malignant pancreatic cases with 100% accuracy.^[42]^ Furthermore, Naito et al. assessed PDAC using FNB-based slides with a CNN, achieving an AUC (area under the receiver operating characteristic curve) of 0.984,^[43]^ whereas Kurita et al. combined biomarkers, cytological features, and clinical variables to differentiate malignant from benign pancreatic cystic lesions using AI, surpassing traditional methods in sensitivity and accuracy.^[44]^

Fu et al. introduced a CNN architecture for classifying and segmenting PDAC in WSI datasets, with notable accuracy and Dice coefficient performances.^[45]^ Other studies, such as those by Chang et al. and Song and Lee, have applied AI to classify nuclei and grade pancreatic adenocarcinoma based on morphological features, demonstrating high accuracy in binary classification.^[46,47]^ Langer et al. identified early pancreatic lesions in mice with a 93% success rate using feature analysis in model training.^[48]^ Meanwhile, Le et al. explored noisy label classification methods for predicting areas of pancreatic adenocarcinoma in WSIs, highlighting the challenges in feature-based classification and the extraction of relevant pathological characteristics.^[49]^

Our research indicated the viability of using an AI-based approach to segment PDAC tissue from WSIs. Furthermore, we explored different network designs that have not been previously applied to PDAC WSI segmentation. To the best of our knowledge, the U-Net++, (SE) U-Net, and Inception U-Net architectures have not been before utilized for PDAC segmentation in EUS-guided FNB samples based on WSI. Our results showed that by increasing the complexity and running time of a number of the U-Net variants, their performance was greater (Inception U-Net and Dense U-Net variants). Among the U-Net variants with less complexity and running time, the Vanilla U-Net had excellent performance for both datasets.

Certainly, in real-world applications for clinical practice, it is crucial to take into account factors such as training time, evaluation speed, and model complexity. In some scenarios, marginal gains in performance may not justify the trade-off of heightened complexity and diminished speed. For instance, although the Vanilla U-Net stands out as the quickest to train and evaluate, boasting the fewest parameters and thus lower complexity, its performance remains on par and competitive with the majority of alternative architectures across the 2 datasets. Also, for the analyzed Dataset 1, Residual U-Net performs slightly better than Vanilla U-Net and comparable in terms of parameters, training and evaluation time. Interestingly, the performance of some modified U-Net variants (SE-U-Net, U-Net++, Attention U-Net) did not surpass that of the standard Vanilla U-Net. The poorest results for both datasets were obtained by SE-U-Net architecture [Table 3, Table 4].

Based on our results, there is clear a trade-off between memory, training time, evaluation time, and performance. However, we observed improvements in performance across both datasets and models, suggesting that more complex, but slower models are the optimal choices for PDAC WSI segmentation. Moreover, the limited availability of publicly accessible datasets containing pancreatic histopathological images has resulted in a scarcity of research on the automated detection of PDAC, especially based on WSI. Evaluating the comparative performance of deep learning algorithms in PDAC WSI segmentation is challenging due to the absence of comprehensive studies and the lack of methods matching in previous assessments. Additionally, variations in datasets across different studies further complicate the assessment of relative performance.

Although AI has made notable advancements in pathological diagnosis, there is limited research examining its capabilities in recognizing and localizing lesions during EUS-FNA/B.^[50]^ Fu et al.^[45]^ utilized base Vanilla U-Net architecture to predict and locate the PDAC regions in WSIs. The dataset contained 1732 patches of 1024 × 1024 pixels corresponding to annotated PDAC areas from 6 WSIs. The patch-level segmentation model had a performance of 84.65% for Dice coefficient on a test dataset containing 163 patches. Instead of focusing on segmenting PDAC regions in EUS-FNB WSIs, another study aimed to train a deep learning model for the classification of PDAC in EUS-FNB WSIs.^[43]^ The authors used a large dataset for developing the model consisting of 532 WSIs from Kurume University. Their model also showed excellent results, with an AUC of 0.9836, accuracy of 0.9417, F1 score of 0.9581, sensitivity of 0.9302, and specificity of 0.9706.

The development of hardware and software capabilities and the demand for clinical services increases lead to notable progress in leveraging AI for the interpretation of images in EUS-FNA/B. In pancreatic EUS-FNA/B, AI primarily contributes to pathological diagnosis and for real-time guidance during puncture procedures.^[50]^ The characteristic of EUS-FNB to collect larger tissue samples, while preserving the architectural features of the tissue, enhances the accuracy of diagnosis for pancreatic lesions.^[51]^ Although the present findings showed promise, it is essential to acknowledge several limitations in our study. Future enhancements can be made in the areas of segmentation performance and overall generality.

Our study has several limitations. First, for a better generalization, we will include more medical centers, with a multicentric prospective protocol. Also, important improvement can be obtained if each tissue section is evaluated and classified by multiple experts, and the study could benefit from a consensus diagnosis from different experts. Beyond the developing of models with improved performance and generalizability through the utilization of larger datasets, the upcoming research endeavors will focus on refining data preparation and optimizing the AI algorithms. The quality of the ground truth data could be enhanced by including the immunohistochemistry-stained sections that provide better contrast than the hematoxylin and eosin–stained sections. The immunohistochemistry-stained sections can improve the identification of mislabeled cells and automatically generate annotations that follow the contours of structures more precisely. For the optimization of AI algorithms, we will take into consideration other methods such as ensemble learning techniques and segmentation approaches based on active or semisupervised techniques.

## CONCLUSION

In this study, we have performed a comparison of 7 U-Net architectures for the automatic detection of PDAC in EUS-FNB samples based on whole-slide imaging. The tested U-Net architectures provided excellent results for PDAC histological image segmentation and suggested that there were differences in performance between U-Net models, using the mean Dice coefficient and mean IoU as evaluation metrics.

## Data Availability

The data will be available upon request to authors and under restrictions regarding ethical aspects.
